# Associations between cognitive reserve, iron deposition in the basal ganglia, and cognitive function in mild cognitive impairment

**DOI:** 10.3389/fpsyg.2026.1900685

**Published:** 2026-09-18

**Authors:** Liang Zhou, Yang Liu, Zhongkai Xie, Juan Li, Huanwei Cheng, Jingxian Shi, Sibin Liu, Wenze Wu, Shuhua Luo

**Affiliations:** 1 Department of Radiology, Jingzhou Hospital Affiliated to Yangtze University, Jingzhou, China; 2 Institute of Medical Imaging, Jingzhou Central Hospital, Jingzhou, China; 3 Ganzhou Institute of Medical Imaging, Medical Imaging Center, Ganzhou People’s Hospital, Ganzhou Hospital-Nanfang Hospital, Southern Medical University, Ganzhou, China

**Keywords:** cognitive function, cognitive reserve, iron deposition, mild cognitive impairment, quantitative susceptibility mapping

## Abstract

**Objective:**

This research aims to investigate the putative mechanisms underlying the association between iron deposition and cognition in mild cognitive impairment (MCI). It also seeks to analyze whether the relationship between cognitive reserve (CR) and cognition may be mediated by iron deposition.

**Methods:**

In total, 109 individuals with MCI and 90 healthy controls (HCs) were enrolled to evaluate iron deposition in the basal ganglia using quantitative susceptibility mapping (QSM). The Cognitive Reserve Index questionnaire (CRIq) was used to evaluate the comprehensive CR of participants in both groups. QSM values in the basal ganglia were compared between the two groups, and Spearman correlation analyses were performed to examine the associations between CR, QSM values, and cognition. Mediation analyses using a bootstrapping approach were conducted to investigate the correlations between CR, QSM values, and cognition.

**Results:**

The MCI group exhibited significantly higher QSM values in the bilateral putamen (PU), substantia nigra (SN), red nucleus (RN), and right caudate nucleus (CA) compared to HCs. After adjusting for age and sex, QSM values in the left RN were negatively correlated with Montreal Cognitive Assessment (MoCA), Mini-Mental State Examination (MMSE), Auditory Verbal Learning Test (AVLT) long-delay recall, and AVLT recognition scores. In contrast, QSM values in the bilateral RN and left SN were negatively correlated with Clock Drawing Test (CDT) scores in the MCI group. CRIq scores were significantly negatively correlated with QSM values in the bilateral RN and right SN. Meanwhile, QSM values in the bilateral RN partially mediated the association between the CRIq scores and cognition in the MCI group.

**Conclusion:**

Individuals with MCI who have different CR levels exhibited differences in iron deposition in the basal ganglia. Comprehensive CR may delay the decline of cognitive function by regulating iron deposition in the bilateral RN during the MCI stage.

## Introduction

Mild cognitive impairment (MCI) refers to a transitional stage between normal aging and dementia, characterized by mild cognitive decline that does not meet dementia diagnostic criteria and does not affect daily living (Jia et al., 2020). As a prodromal stage of Alzheimer’s disease (AD), MCI is widely recognized as a “window of opportunity” for intervening in AD progression, during which timely interventions may prevent or delay dementia onset and slow the progression of pathological damage (Dhakal et al., 2025). Therefore, the early identification of MCI, along with prompt diagnosis and targeted interventions, could serve as a pivotal strategy to attenuate and prevent the progression of AD to irreversible brain damage. According to the National Institute on Aging and Alzheimer’s Association (NIA–AA) research framework for AD (Jack et al., 2018), β-amyloid (Aβ) deposition and tau-mediated neurofibrillary tangles constitute the core pathological hallmarks of AD, while neurodegeneration and brain iron dysregulation represent important co-pathological features that interact with core pathologies to drive cognitive decline. Several reports have demonstrated that AD patients exhibit not only core pathophysiological changes but also excessive iron deposition and metabolic disorders in brain tissue, both of which are associated with AD-related neurodegeneration (Peng et al., 2021).

Neuropathological investigations have confirmed a clear association between brain iron deposition and accelerated cognitive decline in individuals with AD and MCI. Iron levels in specific brain regions (such as the basal ganglia) tend to increase with age, and age-related iron accumulation also occurs in the prefrontal cortex, occipital cortex, and hippocampus—regions integral for cognitive and visual functioning (Burgetova et al., 2021; Black et al., 2023).

Quantitative susceptibility mapping (QSM) quantitatively analyzes iron content by assessing tissue susceptibility and is currently the primary *in vivo* method for measuring brain iron levels (Chen et al., 2025). Accumulating evidence indicates that cognitive decline correlates significantly with QSM values across distinct brain regions during AD progression, highlighting the diagnostic potential of regional iron levels in MCI for early AD detection(Yang et al., 2022). Recent studies have consistently shown that the MCI group exhibits elevated QSM values in multiple brain regions compared to healthy controls (HCs), especially in the basal ganglia (Li et al., 2020) and thalamus (Qin et al., 2022). These values display a significant positive correlation with cognitive impairment (Yu et al., 2023; Zhang et al., 2026).

The concept of cognitive reserve (CR) was proposed to address the key question of why individuals exhibit different levels of cognitive performance despite having similar levels of brain pathology, and it has since become a core framework for understanding resilience against age-related cognitive decline (Stern and Barulli, 2019). CR refers to the brain’s protective capacity against damage from pathological conditions, influenced by lifestyle factors accumulated throughout a person’s lifespan. According to CR theory, individuals can optimize and maximize brain function via structural and network remodeling to counteract cognitive deficits induced by age-related cognitive decline and pathological insults (Stern, 2012). Previous studies have underscored the potential role of CR in healthy and pathological aging, with its protective effect against dementia development being particularly prominent (Menardi et al., 2018; Nelson et al., 2021; Han et al., 2025). Accumulating evidence confirms that improving educational levels and implementing life skills training can enhance CR, reduce MCI risk, and delay the onset and progression of clinical cognitive decline (Livingston et al., 2020; Corbo et al., 2023).

Emerging evidence indicates that cerebral iron homeostasis is dynamically modulated by modifiable lifestyle factors and vascular health status (Gustavsson et al., 2024). Deteriorated vascular function accelerates pathological brain iron accumulation, whereas healthy dietary and behavioral lifestyles can effectively alleviate age-related iron deposition and mitigate working memory decline (Zachariou et al., 2021). Notably, previous studies have demonstrated that gray matter atrophy and excessive brain iron accumulation are closely associated with cognitive impairment in patients with subcortical vascular mild cognitive impairment (svMCI) (Liu et al., 2025). Despite growing evidence linking CR to cognitive resilience in MCI and iron metabolism to AD pathogenesis, existing MRI studies investigating the neural mechanisms underlying CR in MCI have mainly focused on structural and functional brain alterations. The specific effect of comprehensive CR on basal ganglia iron deposition in MCI remains unexplored, and the potential mediating role of CR in the relationship between brain iron metabolism and cognitive function in MCI remains elusive. Therefore, this study aimed to investigate the effects of comprehensive CR on iron deposition in the basal ganglia of individuals with MCI using QSM. Specifically, we first compared QSM values in the basal ganglia between the MCI and HC groups to identify regions with significant differences. Subsequently, we examined the relationships between QSM values in these regions, CR, and cognitive function, and a mediation analysis was conducted to examine the association between CR, QSM values, and cognitive function. We hypothesized that individuals with MCI who have different CR levels may exhibit distinct QSM values in the basal ganglia and that these region-specific iron accumulation patterns may correlate with cognitive performance.

## Materials and methods

### Participants

A total of 109 individuals with MCI and 90 HCs, matched for age and sex, were enrolled in this study. The MCI group was primarily recruited from the Departments of Neurology and Geriatrics and the Memory Clinic, and they reported experiencing subjective memory decline. The HC group consisted of older community residents and family members of individuals with partial MCI. This study was approved by the Ethics Committee of Jingzhou Hospital affiliated with Yangtze University. All participants were fully informed about the entire research protocol and provided written informed consent before enrollment.

### Inclusion criteria for the MCI and HC groups

The inclusion criteria for the MCI group were as follows: (1) age between 50 and 80 years; (2) meeting the diagnostic criteria for MCI proposed by Petersen (2011); (3) objective impairment in one or more cognitive domains, as confirmed by neuropsychological assessment, without meeting the diagnostic criteria for dementia; (4) a score of 0.5 on the Clinical Dementia Rating (CDR) scale; (5) preserved activities of daily living; and (6) right-handedness. The inclusion criteria for the HC group were as follows:(1) age between 50 and 80 years; (2) good general physical condition, with no severe dysfunction of vital organs, malignant tumor, uncontrolled hypertension or diabetes, history of psychiatric disorders, or alcohol or substance dependence, and the ability to fully cooperate with the complete MRI scan and neuropsychological assessment; (3) normal global cognitive function, with a Mini-Mental State Examination (MMSE) score ranging from 27 to 30; (4) no subjective memory loss or other cognitive complaints; and (5) right-handedness.

### Demographic data and neuropsychological assessment

Demographic information, cognitive function measures, and CR data were collected from all participants on the same day as QSM image acquisition. Basic demographic data were collected from all the enrolled participants. According to the application guidelines for the Chinese Brief Cognitive Test in dementia diagnosis, global cognitive function was assessed using the Montreal Cognitive Assessment (MoCA) and MMSE. The Auditory Verbal Learning Test-Huashan version (AVLT-H) was used to evaluate episodic memory function, including immediate recall, short-delay recall (5 min), long-delay recall (20 min), and recognition. Language function was measured using the Verbal Fluency Test (VFT). The Clock Drawing Test (CDT) was used to assess visuospatial ability and executive function. The Digit Span Test (DST) was used to evaluate auditory memory span and digital processing capacity. Daily living status was evaluated using the Activities of Daily Living (ADL) and Instrumental Activities of Daily Living (IADL) scales. The 15-item Geriatric Depression Scale (GDS) was utilized to assess depressive symptoms in both groups.

### CR assessment

The Cognitive Reserve Index questionnaire (CRIq) (Nucci et al., 2012) was used to evaluate the comprehensive CR of participants in both groups. The CRIq integrates three dimensions: educational attainment, occupational achievement (after 18 years of age), and daily leisure activities (after 18 years of age), with a total score calculated using a built-in weighted formula. The CRIq education domain consists of years of schooling plus the duration of occupation-related training, with 1 point awarded for each full year and 0.5 points for periods exceeding 6 months; periods shorter than 6 months receive no points. The CRIq occupation domain divides occupations into five hierarchical levels: unskilled manual labor (Level 1; e.g., farmers, nannies, and waiters), skilled manual labor (Level 2; e.g., chefs, tailors, and factory workers), skilled non-manual/technical work (Level 3; e.g., white-collar workers, accountants, and self-employed individuals), professionals (Level 4; e.g., lawyers, teachers, and physicians), and senior intellectuals (Level 5; e.g., civil servants, senior managers, and university professors). The duration of each occupation is calculated in 5-yea*r* units, rounded to the nearest multiple of 5 years (e.g., 12 years is rounded to 15 years, and 17 years is rounded to 20 years). Scores from different occupations across age groups can be accumulated; the occupational score is calculated by multiplying the duration of employment by the corresponding occupation level. The CRIq leisure activities domain categorizes activities by frequency: weekly (e.g., reading newspapers/magazines, doing housework, participating in sports, dancing, and playing chess), monthly (e.g., attending gatherings, watching movies, singing, and caring for family members), annual (e.g., long-distance travel, attending conferences or concerts, and reading books), and fixed routine activities (e.g., pet care, financial management, and number of children). Only activities performed more than 3 times per cycle and lasting over 1 year were scored, with duration calculated using the same rounding rule as that applied to occupational duration. Detailed instructions and scoring criteria are available at http://www.cognitivereserveindex.org. A higher total CRIq score indicates better CR. The CRIq has been validated in the Chinese population, demonstrating favorable internal consistency and validity (Cao et al., 2022).

### QSM data acquisition and processing

All multimodal MRI data were acquired using a Philips Ingenia CX 3.0 T superconducting MRI scanner (Philips Healthcare, Best, the Netherlands) at the Department of Magnetic Resonance Imaging, the Second Hospital of Lanzhou University, equipped with a 32-channel phased-array head coil. QSM data were acquired using a 3D multi-echo gradient-recalled echo (GRE) sequence, and QSM reconstruction was performed based on the obtained T2* data. The detailed scanning parameters were as follows: repetition time (TR) = 55 ms, echo time (TE) = 5.10 ms, flip angle = 12°, slice thickness = 2 mm, number of slices = 64, field of view (FOV) = 256 mm × 256 mm, and matrix = 256 × 256.

Before data processing, two radiologists independently performed a visual inspection of all whole-brain QSM, magnitude, and phase images. Participants with severe motion artifacts, global phase unwrapping failures, or extensive signal distortion caused by major susceptibility artifacts were excluded during the initial enrollment stage. QSM data processing and analysis were conducted using STI Suite v3.0 (https://people.eecs.berkeley.edu/~chunlei.liu/software.html), which is an open-source MATLAB-based toolbox for QSM. The processing pipeline was performed as follows: Phase unwrapping was conducted using the Laplacian-based phase unwrapping algorithm (Li et al., 2011). A brain mask was generated from the middle-echo magnitude image. First, the middle-echo magnitude map was squared and lightly smoothed using a Gaussian filter, followed by optional correction for intensity inhomogeneity using N4 bias field correction. The FSL BET algorithm with fixed parameters (−f 0.3, −R) was applied to generate a coarse brain mask. The subsequent postprocessing included the extraction of the largest connected component, filling of intracranial holes using 3D morphological closing, one-voxel edge erosion, and secondary intensity thresholding constrained by the middle-echo magnitude to eliminate low-SNR voxels at the brain boundaries. Spatial filtering was applied to the phase data using the variable-kernel sophisticated harmonic artifact reduction for phase data (V-SHARP) method, implemented in MATLAB, to remove background fields and calculate local field maps (Wu et al., 2012). Streaking artifact removal was performed to generate the final QSM images (Wei et al., 2015).

Regions of interest (ROIs) encompassing bilateral gray matter nuclei were manually delineated on the susceptibility maps using ITK-SNAP software. The delineated regions included the caudate nucleus (CA), globus pallidus (GP), putamen (PU), dorsal thalamus (DT), red nucleus (RN), and substantia nigra (SN). The mean QSM value for each ROI was extracted for subsequent analysis (Figure 1). To ensure repeatability, the intraclass correlation coefficient (ICC) was calculated between the two analysts, and only results with an ICC > 0.80 were included in the final analysis. Both analysts were blinded to the diagnosis and CR status during the manual segmentation.

**Figure 1:**
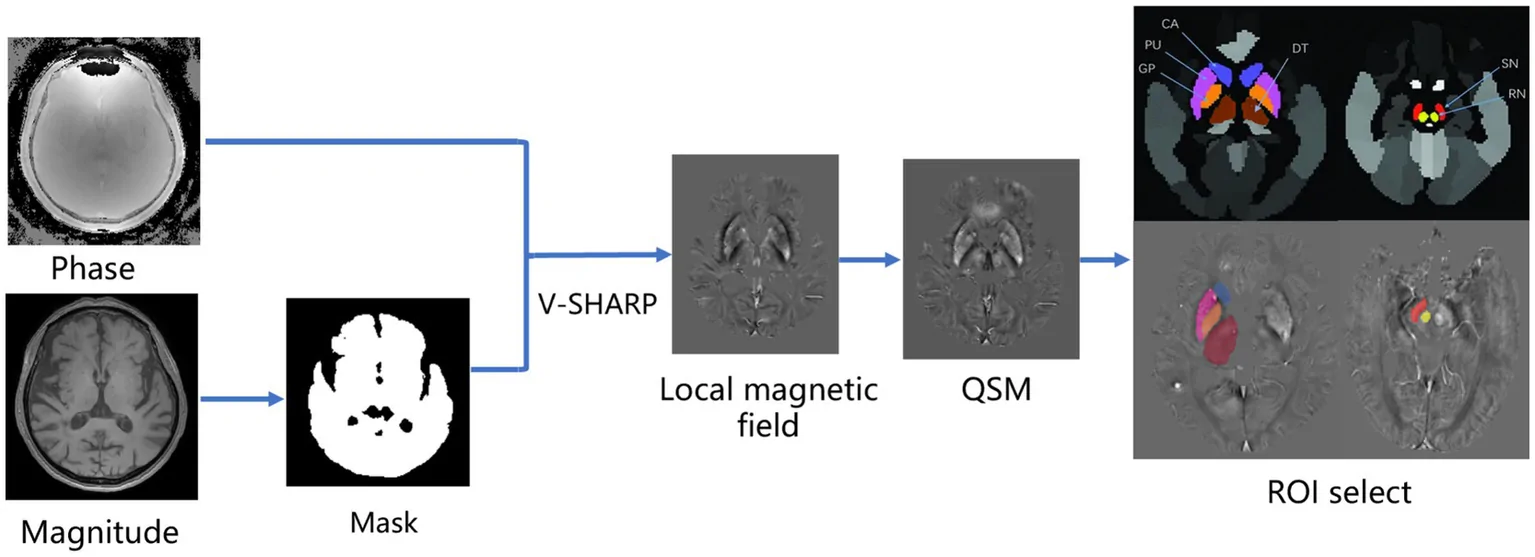
Simplified flowchart of QSM image processing.

### Statistical analysis

All statistical analyses were performed using SPSS 26.0 (SPSS, Chicago, IL, USA). Levene’s test was used to assess the normality and homogeneity of variance of quantitative data. Quantitative data with a normal distribution and homogeneous variance were presented as mean ± standard deviation (SD), and between-group comparisons were performed using independent-samples *t*-tests. Non-normally distributed continuous data were expressed as medians (interquartile ranges) [M (P25, P75)], and between-group differences were analyzed using the Mann–Whitney U-test. Brain regions with significant QSM differences between the two groups were selected as ROIs, and their QSM values were extracted for follow-up statistical analysis. Spearman’s correlation analysis was conducted to examine the associations between QSM values, CRIq scores, and clinical neuropsychological scale scores. Furthermore, a bootstrapping approach to mediation analysis, as proposed by Preacher and Hayes (2008), was conducted with 5,000 iterations to analyze the relationships between CRIq, QSM values, and cognitive scale scores. This method generates 95% confidence intervals (CIs) for the mediation effect; a CI that does not contain 0 indicates a significant mediation effect. Mediation analysis was performed using the SPSS plug-in PROCESS (version 4.1, https://www.processmacro.org/download.html). A two-tailed *p*-value of < 0.05 was considered statistically significant. For analyses involving multiple comparisons, *p*-values were adjusted using the Benjamini–Hochberg false discovery rate (FDR) procedure, with an FDR-adjusted *p*-value of < 0.05 considered significant for the primary analyses, including between-group comparisons and correlation analyses.

## Results

### General demographic and clinical characteristics

A total of 199 participants, comprising 109 individuals with MCI and 90 HCs, were included in the final analysis. Among the 109 MCI participants, 83 were classified as having amnestic MCI, including 40 cases with single-domain memory impairment and 43 cases with multi-domain impairment (memory plus executive/visuospatial functions). The remaining 26 participants were classified as having non-amnestic MCI, predominantly presenting with executive function or visuospatial impairment. Given the uneven and relatively small sample sizes across subgroups, formal statistical analyses of subgroup differences were not performed in the current study. The baseline demographic characteristics and clinical cognitive scores of the two groups are presented in Table 1. No significant between-group differences were observed for age (*p* = 0.11), sex (*p* = 0.457), ADL scores (*p* = 0.962), or GDS scores (*p* = 0.109). The HC group presented markedly higher CR scores than the MCI group, and significant between-group differences were observed for all cognitive scale scores (all *p* < 0.05), except for ADL and GDS scores (Table 1).

**Table 1:** Demographic characteristics between the HC and MCI groups.

Characteristics	HC (*n* = 90)	MCI (n = 109)	*P* (FDR-adjusted)
Age (y)	61.07 ± 0.53	62 (58–68)	0.11
Sex (m/f)	30/60	43/66	0.457
Education (y)	11 (10–14)	8 (5–10)	<0.001
CRIq	100 (89–110)	85 (77–93)	<0.001
CRIq education	101.17 (11.19)	91 (79–99)	<0.001
CRIq occupation	111 (91–123.75)	91 (87–100)	<0.001
CRIq leisure	82 (81–98.75)	82.46 (8.63)	<0.001
MoCA	27 (26,28)	21 (18–23)	<0.001
MMSE	28 (28,29)	25 (23–26)	<0.001
AVLT immediate recall	6 (5–7)	4 (3–5)	<0.001
AVLT short-delay recall	7 (5.25–7)	3 (2–4)	<0.001
AVLT long-delay recall	6 (5–7)	3 (2–4)	<0.001
AVLT recognition	21 (21–22)	19 (17–20)	<0.001
DST	12.5 (12–13.75)	10 (9–11)	<0.001
VFT	17 (14–21)	13 (12–16)	<0.001
CDT	4 (4–4)	3 (3–4)	<0.001
ADL	8 (8–8)	8 (8–8)	0.962
GDS	1 (1–1)	1 (1–2)	0.109

Normal distribution data are presented as mean (SD); non-normal distribution data are presented as median (interquartile range); HC, healthy controls; MCI, mild cognitive impairment; CRIq, cognitive reserve index questionnaire; MoCA, Montreal cognitive assessment; MMSE, mini-mental state examination; AVLT, auditory verbal learning test; CDT, clock drawing test; VFT, verbal fluency test; DST, digital span test; ADL, activity of daily living; GDS, geriatric depression scale.

Normally distributed data are presented as mean (SD), whereas non-normally distributed data are presented as median (interquartile range). HC, healthy controls; MCI, mild cognitive impairment; CRIq, Cognitive Reserve Index questionnaire; MoCA, Montreal Cognitive Assessment; MMSE, Mini-Mental State Examination; AVLT, auditory verbal learning test; CDT, clock drawing test; VFT, verbal fluency test; DST, digital span test; ADL, Activities of Daily Living; GDS, Geriatric Depression Scale.

### Comparison of QSM values in the basal ganglia between the HC and MCI groups

Only results with ROI-specific ICC values > 0.80 were included in the final analysis (all >0.85: RN 0.92, SN 0.89, PU 0.87, and CA 0.86). The MCI group exhibited significantly higher QSM values in the bilateral PU, SN, RN, and right CA compared to the HC group (all *p* < 0.05; Table 2; Figure 2). No significant differences were observed for QSM values in the left CA, bilateral GP, and DT (all *p* > 0.05) between the two groups.

**Table 2:** QSM values in the HC and MCI groups/×10^−3^ ppm.

Region	HC (*n* = 90)	MCI (*n* = 109)	*P* (FDR-adjusted)
Left CA	10.2 ± 9.9	11.1 (3.7–16.4)	0.626
Right CA	5.6 (0.4–12.4)	7.7 (3.1–15.4)	0.037
Left GP	54.9 (48–67.8)	58.5 (50.2–69.9)	0.245
Right GP	57.8 (47.9–72.4)	59.1 (50–67.7)	0.553
Left PU	36.6 (29–46.3)	43.4 (35.9–51.2)	0.001
Right PU	34 (24.5–41.4)	39.9 (33.5–48.1)	<0.001
Left DT	4.3 (1.6–8.4)	4.4 (0.8–7.3)	0.451
Right DT	4.1 (2.2–7.6)	5.7 (3.2–9.3)	0.095
Left RN	36.6 (29.5–45.7)	42.9 (34.1–58.3)	0.004
Right RN	40 (30.7–49.4)	45.2 (34.9–60.1)	0.004
Left SN	58 (45.2–68.6)	65 (52.6–76.2)	0.002
Right SN	51.4 (43.3–61.6)	60.2 (49.9–68.5)	0.002

Normal distribution data are presented as mean ±SD; non-normal distribution data are presented as median (interquartile range); HC, healthy controls; MCI, mild cognitive impairment; CA, caudate nucleus; GP, globus pallidus; PU, putamen; DT, dorsal thalamus; RN, red nucleus; SN, substantia nigra.

**Figure 2:**
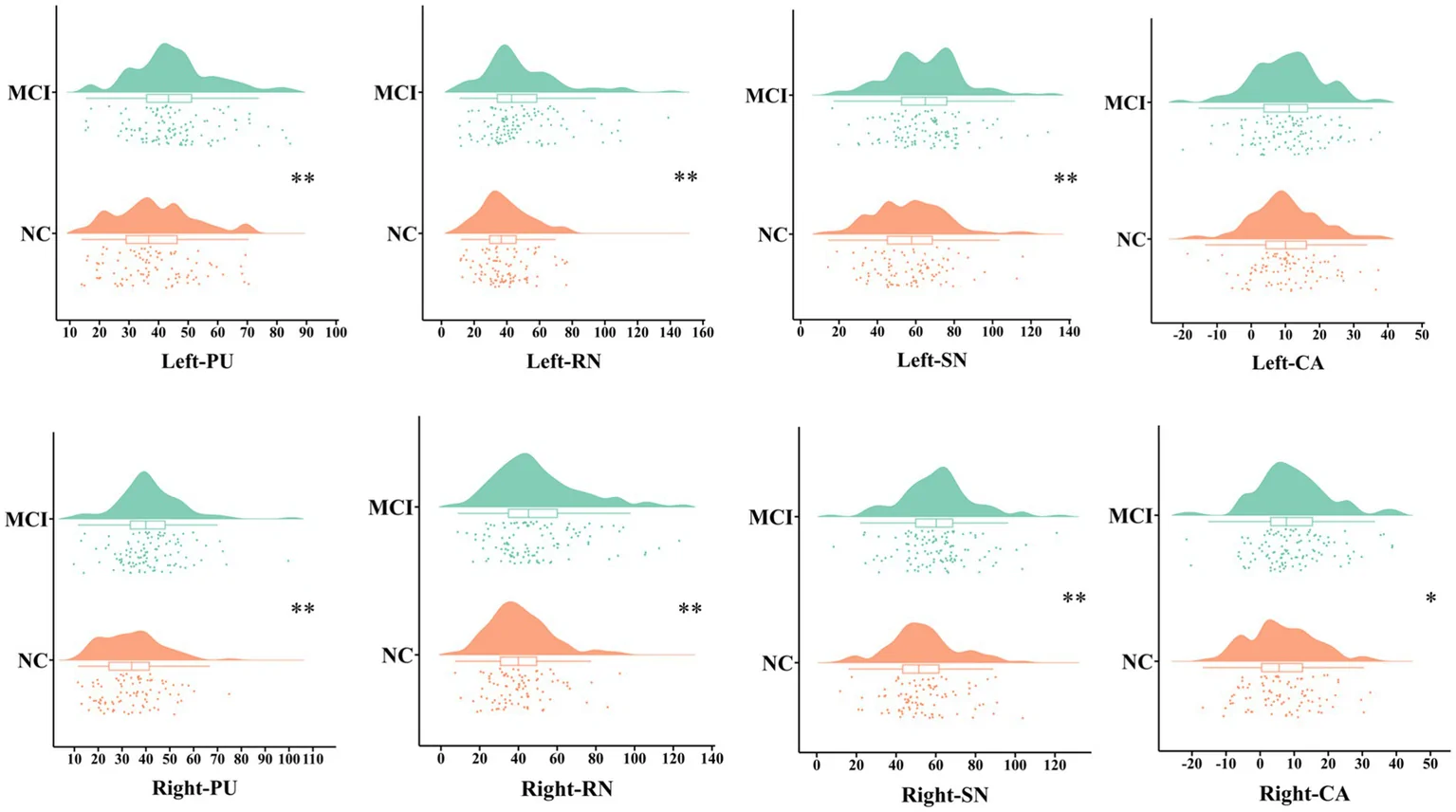
Comparison of QSM value distributions in the basal ganglia between the HC and MCI groups (×10^−3^ p_pm_). HC, healthy controls; MCI, mild cognitive impairment; CA, caudate nucleus; PU, putamen; RN, red nucleus; SN, substantia nigra; *p < 0.05; **p < 0.01.

Normally distributed data are presented as mean ±SD, while non-normally distributed data are presented as median (interquartile range). HC, healthy controls; MCI, mild cognitive impairment; CA, caudate nucleus; GP, globus pallidus; PU, putamen; DT, dorsal thalamus; RN, red nucleus; SN, substantia nigra.

### Correlation between QSM values and cognitive function in the MCI Group

Brain regions showing significant between-group differences in QSM values were selected as ROIs. Spearman correlation analysis was performed to examine the associations between QSM values in these ROIs and cognitive scale scores. Given that brain iron concentration is positively correlated with age and differs by sex in elderly individuals (Grubić Kezele and Ćurko-Cofek, 2020), a partial Spearman correlation analysis was conducted with age and sex as control variables. After controlling for age and sex, QSM values in the left RN were negatively correlated with MoCA (*r* = −0.209, *p* = 0.029), MMSE (*r* = −0.187, *p* = 0.031), AVLT long-delay recall (*r* = −0.182, *p* = 0.033), and AVLT recognition scores (*r* = −0.204, *p* = 0.035). QSM values in the bilateral RN (left: *r* = −0.243, *p* = 0.01; right: *r* = −0.222, *p* = 0.021) and left SN (*r* = −0.197, *p* = 0.042) were negatively correlated with CDT scores (Figure 3).

**Figure 3:**
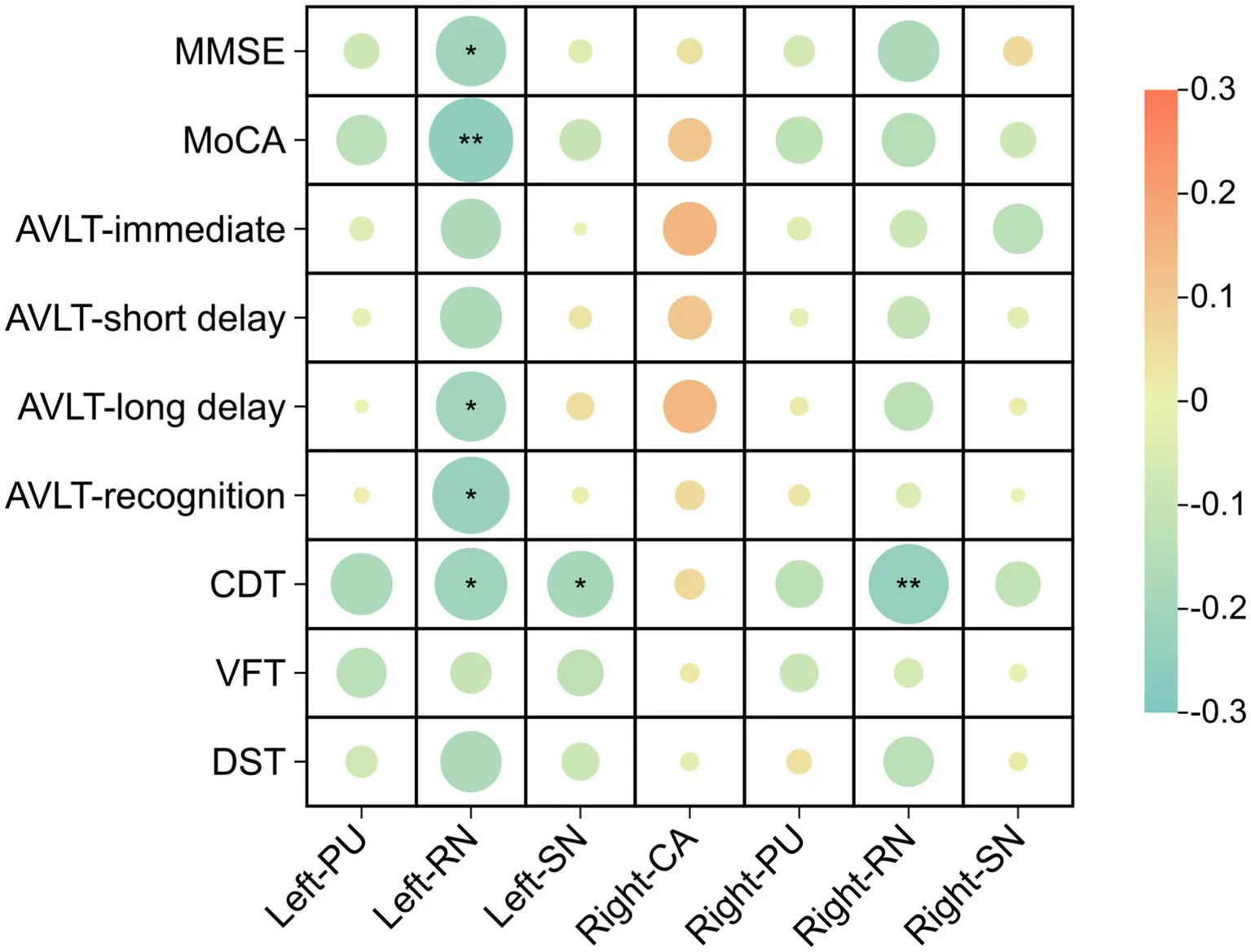
Partial correlations between QSM values and cognition in the MCI group (FDR-adjusted). MoCA, Montreal Cognitive Assessment; MMSE, Mini-Mental State Examination; AVLT, auditory verbal learning test; CDT, clock drawing test; VFT, verbal fluency test; DST, digital span test; CA, caudate nucleus; PU, putamen; RN, red nucleus; SN, substantia nigra; *p < 0.05; **p < 0.01.

### Correlation between QSM values and CR in the MCI Group

Spearman correlation analysis was performed to examine the associations between CRIq scores and QSM values in the basal ganglia nuclei, which were significantly higher in the MCI group compared to the HC group. After adjusting for age and sex in the partial correlation analysis, CRIq scores were significantly negatively correlated with QSM values in the bilateral RN (left: *r* = −0.278, *p* = 0.003, Figure 4A; right: *r* = −0.202, *p* = 0.035, Figure 4B) and right SN (*r* = −0.285, *p* = 0.002, Figure 4C).

**Figure 4:**
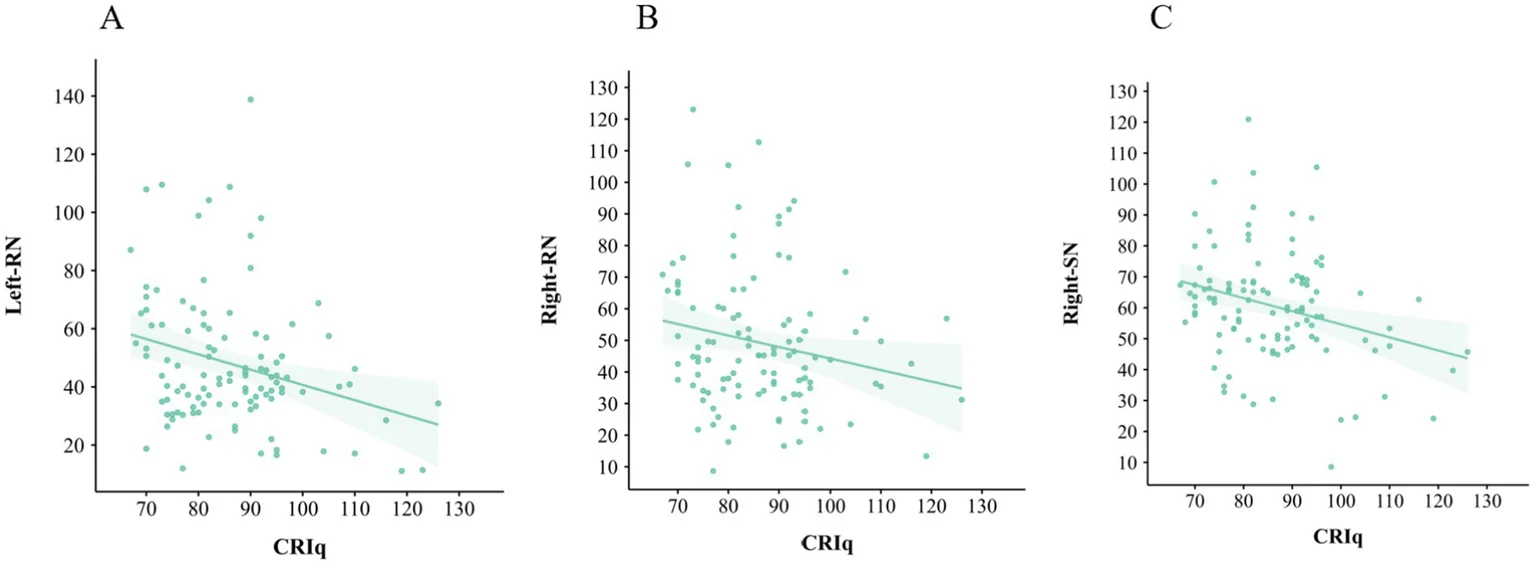
Scatter plot of the partial correlation between QSM values (×10^−3^ ppm) and CRIq scores in the MCI group (FDR-adjusted). CRIq, Cognitive Reserve Index questionnaire; RN, red nucleus; SN, substantia nigra.

### Mediation analysis

Individuals with higher CR exhibited lower QSM values within the MCI group. Therefore, we hypothesize that CR may play an important role in the progression of MCI. A mediation analysis was performed with age and sex as covariates, cognitive scores as dependent variables, CRIq as the independent variable, and the bilateral RN as the mediator.

In the MCI group, QSM values in the left RN partially mediated the association between CRIq and MoCA scores, with the mediation effect (a × b) accounting for 9.1% of the total effect (c) (Figure 5A). QSM values in the bilateral RN partially mediated the association between CRIq and CDT scores, with mediation effects accounting for 25.1% (left RN, Figure 5B) and 16.2% (right RN, Figure 5C), respectively.

**Figure 5:**
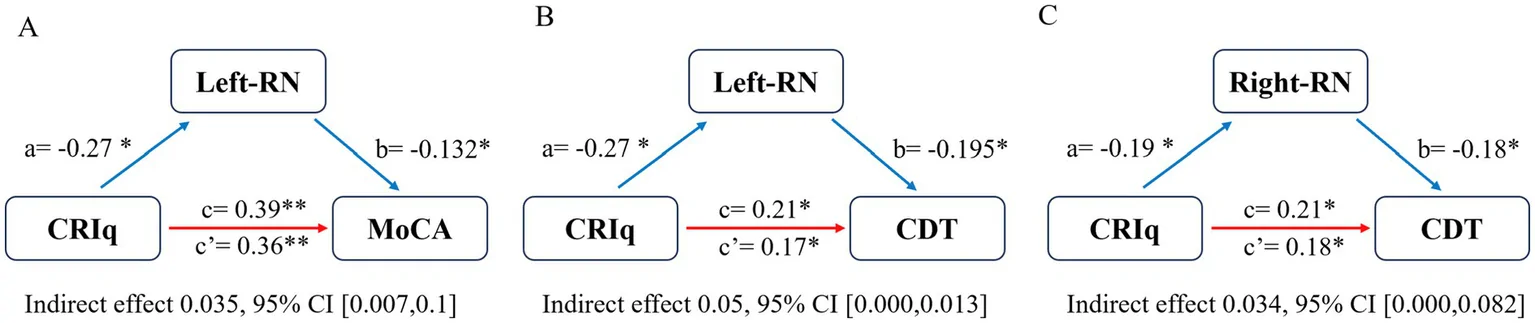
Simple mediation analysis of the effects of QSM values on the relationship between CR and cognitive function in the MCI group; **(A–C)**, and *c*’ are path coefficients representing unstandardized regression weights and standard errors (in parentheses). The *c* path coefficient represents the total effect. The *c*’ path coefficient refers to the direct effect. All analyzed *a*, *b*, *c*, and *c*’ paths were significant (*p < 0.05, **p <0.001). CRIq, cognitive reserve index questionnaire; RN, red nucleus; MoCA, Montreal Cognitive Assessment; CDT, clock drawing test.

## Discussion

This study analyzed the role of comprehensive CR in iron deposition within the basal ganglia nuclei in individuals with MCI. Our core findings are threefold: (1) individuals with MCI exhibited significantly higher iron deposition in the PU, SN, RN, and right CA compared to HCs; (2) higher CR was independently associated with reduced iron deposition in the bilateral RN; and (3) bilateral RN iron deposition partially mediated the association between CR and cognitive performance. These findings provide novel cross-sectional evidence that CR is associated with reduced iron deposition in the RN and that RN iron deposition partially mediates the association between CR and cognitive performance.

Excessive brain iron accumulation is a well-recognized pathological feature of aging and neurodegenerative diseases. Iron overload generates reactive oxygen species via the Fenton reaction, leading to mitochondrial dysfunction, neuronal apoptosis, and synaptic plasticity impairment, all of which contribute to cognitive decline (Smith et al., 1997; Zecca et al., 2004). Recent comprehensive reviews have confirmed that brain iron dysregulation is not merely a secondary phenomenon but an active driver of cognitive impairment across the AD spectrum, with iron deposition colocalizing with β-amyloid plaques and tau aggregates and exacerbating pathological progression (Peng et al., 2021; Levi et al., 2024; Chen et al., 2025). Our results are consistent with previous studies showing elevated QSM values in multiple deep gray matter regions of individuals with MCI (Li et al., 2020; Yu et al., 2023). Specifically, we found significant iron accumulation in the bilateral PU, SN, RN, and right CA. The basal ganglia are rich in iron under physiological conditions and play critical roles in cognitive regulation, including memory encoding, executive function, and attention (Monchi et al., 2006). Sun et al. (2017) found that QSM values in the bilateral hippocampus and right PU were elevated in patients with svMCI compared to HC and that QSM values in the right PU were negatively correlated with attention-based executive function. In the early stage of MCI, incipient hippocampal atrophy is accompanied by the dysregulation of iron homeostasis (Zhang et al., 2022). Previous studies have demonstrated that the MCI group exhibits elevated QSM values in deep gray matter regions (Yu et al., 2023) compared to HCs, with QSM values showing significant positive correlations with the severity of cognitive impairment (Qin et al., 2022). Notably, we identified the RN as a key region linking iron deposition to cognitive impairment in MCI. After controlling for age and sex, left RN iron deposition correlated with worse global cognition (MoCA and MMSE) and episodic memory (AVLT long-delay recall and recognition), while bilateral RN iron deposition was correlated with impaired executive function (CDT). The RN is a midbrain nucleus with high metabolic activity and iron content, and its iron dyshomeostasis may disrupt subcortical–cortical neural circuits, leading to cognitive deficits (Doyon and Benali, 2005). Our findings extend previous research by highlighting the specific role of RN iron deposition in MCI-related cognitive decline.

A major novelty of this study is the finding that the association between CR and cognitive function in MCI is partially mediated by iron deposition. CR reflects the brain’s ability to compensate for pathological damage through lifelong experiences, including education, occupation, and leisure activities (Stern, 2012). While numerous studies have shown that higher CR reduces MCI risk and slows cognitive decline (Livingston et al., 2020; Iraniparast et al., 2022; Corbo et al., 2023), the underlying biological mechanisms remain poorly understood. We found that higher CR was significantly associated with lower iron deposition in the bilateral RN, even after adjusting for age and sex. The mediation analysis further revealed that bilateral RN iron deposition partially mediated the relationship between CR and cognitive performance, accounting for 4.01–7.59% of the total effect. These results suggest that CR may exert its protective effect, at least in part, by regulating iron metabolism in the RN. The regional specificity of this effect may be attributed to the RN’s high vulnerability to iron-induced oxidative damage, due to its high iron content and metabolic rate (Zecca et al., 2004). As a deep gray matter nucleus with active iron metabolism, the imbalance in iron homeostasis in the RN can lead to an increase in free Fe^2+^, which generates substantial reactive oxygen species. This subsequently causes mitochondrial dysfunction, neuronal apoptosis, and synaptic plasticity impairment. In addition, iron deposition in the RN may also accelerate the pathological progression of AD by promoting the aggregation of α-synuclein or the deposition of β-amyloid (Levi et al., 2024). We hypothesized that the lower bilateral RN susceptibility values observed in MCI individuals with high CR may represent a compensatory protective response to increased brain iron deposition, thereby maintaining iron metabolism activity in MCI. During the transition from normal cognition to MCI, as AD pathology progressively advances, compensatory mechanisms in individuals with lower CR gradually become depleted, ultimately leading to increased QSM values in the bilateral RN. In contrast, MCI individuals with higher CR can resist the pathological changes of AD by reducing QSM values in the bilateral RN.

The mechanisms through which CR modulates brain iron metabolism are likely multifactorial. Individuals with higher CR tend to have healthier lifestyles, including balanced diets, regular physical activity, and better cardiovascular health, all of which have been shown to reduce brain iron accumulation (Zachariou et al., 2021; Gustavsson et al., 2024). For example, dietary antioxidants, such as vitamin C and quercetin, can enhance iron chelation and reduce oxidative stress, while regular exercise improves cerebral blood flow and blood–brain barrier function, facilitating iron efflux (Zachariou et al., 2025). In addition, CR may upregulate the expression of iron regulatory proteins (e.g., hepcidin and transferrin receptor), thereby maintaining iron homeostasis and preventing pathological iron accumulation. On the other hand, recent studies have demonstrated that information transmission capacity within brain structural–functional networks in AD patients is closely associated with iron levels (Liu et al., 2025). QSM values are related to the reduced spatial correlation between global structural and functional efficiencies. Structural and functional hub regions affected by increased iron deposition and atrophy may represent vulnerable regions susceptible to impairment during AD progression. The decline in complex network efficiency in these central regions may contribute to brain dysfunction across diverse cognitive and behavioral domains. Numerous previous studies have demonstrated that CR can better compensate for brain damage in the early stages of AD, specifically the MCI stage, by enhancing the adaptability of neural networks, promoting efficient switching between brain networks, and improving interregional cerebral blood flow connectivity to maintain cognitive function (Colangeli et al., 2016; Brenner et al., 2023; Zhu et al., 2023; Vockert et al., 2024). Therefore, we hypothesized that comprehensive CR may indirectly modulate the spatial correlation between iron metabolism and overall structural–functional efficiency by regulating structural–functional network efficiency in MCI, thereby delaying the cognitive decline induced by iron deposition.

### Limitations of the study

The generalizability of these findings is subject to certain limitations. First, the ROI measurement of QSM values was performed by manual delineation. This measurement may involve certain subjective factors and local susceptibility artifacts (air-tissue interfaces at the skull base/paranasal sinuses, or streaking/shadowing artifacts caused by tiny lacunes in the basal ganglia), even though the measurement was conducted by experienced radiologists using an independent blind method. Second, the study population was relatively small to form subgroups analysis of amnestic MCI and non-amnestic MCI, future studies should extend effect sizes to analysis the stratification value of QSM in subgroups of MCI with different CR. Finally, QSM-based measurements were limited to the basal ganglia, and no measurements were performed in other brain regions. In addition, iron metabolism analysis relied solely on QSM imaging correlations. It should be noted that QSM quantitatively reflects tissue iron deposition levels rather than dynamic iron metabolic processes such as iron transport, storage, and turnover. The hypothesis that CR regulates iron metabolism is based on the observed association and requires further validation using molecular biological approaches, such as ferritinomics and *in vivo* molecular probes. We explicitly acknowledge that the current study cannot establish a temporal or causal relationship between CR, iron deposition, and cognitive decline and that longitudinal studies are needed to determine whether iron deposition mediates the protective effect of CR on long-term cognitive decline.

## Conclusion

In summary, this study demonstrates that individuals with MCI with different CR levels exhibit distinct patterns of basal ganglia iron deposition and that RN iron deposition partially mediates the association between comprehensive CR and cognitive performance. The results suggest that modulation of brain iron homeostasis may represent a putative biological mechanism underlying the protective effect of CR in MCI. Future longitudinal and interventional studies are warranted to validate these findings and explore whether enhancing CR can reduce brain iron deposition and slow cognitive progression in MCI.

## Data Availability

The raw data supporting the conclusions of this article will be made available by the authors, without undue reservation.
